# Providing Feedback on Previous Pain Scores Did Not Affect Weekly Pain Variability: A Cohort‐Nested Randomised Study

**DOI:** 10.1002/ejp.70361

**Published:** 2026-08-20

**Authors:** Casper Nim, Aron Downie, Søren O'Neill, Rikke Krüger Jensen, Sheilah Hogg‐Johnson, Alice Kongsted

**Affiliations:** ^1^ Medical Research Unit, Spine Centre of Southern Denmark University Hospital of Southern Denmark Kolding Denmark; ^2^ Department of Regional Health Research University of Southern Denmark Odense Denmark; ^3^ Department of Chiropractic, Faculty of Medicine, Health and Human Sciences Macquarie University Sydney Australia; ^4^ Center for Muscle and Joint Health, Department of Sports Science and Clinical Biomechanics University of Southern Denmark Odense Denmark; ^5^ Chiropractic Knowledge Hub Odense Denmark; ^6^ Division of Research and Innovation Canadian Memorial Chiropractic College Toronto Canada

**Keywords:** longitudinal monitoring, pain variability, patient‐reported outcomes, spinal pain

## Abstract

**Background:**

Spinal pain is one of the leading causes of disability worldwide and repeated symptom monitoring is increasingly used to capture its fluctuating nature. However, repeated pain assessments may be influenced by prior responses, potentially affecting longitudinal patterns of pain reporting. This study examined whether providing feedback on prior pain scores influenced within‐person variability in weekly pain intensity ratings and retention over 60 weeks.

**Methods:**

This randomised study evaluating a methodological feature of repeated pain assessment was embedded within a cohort of adults with spinal pain referred to an outpatient hospital clinic. Participants (*n* = 2448) were randomised 1:1 to weekly pain intensity ratings (0–10 numerical rating scale) either with feedback (‘You answered [X] last week’) or without feedback. Analyses included participants with ≥ 40% valid responses (*n* = 1883), of whom 948 received feedback and 935 did not. The primary outcome was within‐person variability in pain intensity, quantified using the root mean square of successive differences. Secondary outcomes included additional fluctuation metrics and the number of weeks with missing responses.

**Results:**

No meaningful between‐group differences were observed for the primary outcome (mean difference −0.04 points [95% confidence interval −0.08 to 0.01]) or secondary outcomes, including retention rates. Sensitivity analyses yielded consistent findings.

**Conclusions:**

Providing participants with feedback on their previous pain score did not meaningfully influence within‐person pain variability or retention during 60 weeks of weekly monitoring. These findings aid the interpretation of repeated longitudinal pain assessments by showing that the observed variability was robust to this specific study design.

**Significance:**

This randomised study showed that providing participants with feedback on prior pain scores did not meaningfully alter weekly pain variability or retention during 60 weeks of longitudinal monitoring. These findings contribute to the interpretation of repeated longitudinal pain assessments in spinal pain research and suggest that weekly pain reporting patterns are robust to prior‐pain feedback during long‐term symptom monitoring.

## 1 Introduction

Spinal pain, including low back and neck pain, remains one of the leading causes of years lived with disability worldwide, with prevalence projected to approach one billion people by 2050 (Ferreira et al. 2023; Wu et al. 2024). Spinal pain is a dynamic condition, characterised by recurrent episodes, fluctuating patterns and persistent symptoms with varying pain intensity over time (Kongsted et al. 2016; Hartvigsen et al. 2018). Accordingly, spinal pain research has increasingly moved beyond single time‐point assessments and predefined cut‐points toward repeated longitudinal assessments that capture the course and variability of pain over time (Kongsted et al. 2016; Dunn et al. 2017). Although more frequent symptom assessment provides new opportunities to understand pain trajectories, it also raises methodological questions about how repeated measurement itself may influence longitudinal symptom reporting (Kongsted et al. 2021). Frequent pain intensity assessments have revealed substantial within‐person variability, with fluctuations observed across months (Dunn et al. 2013), weeks (Kongsted et al. 2017; Irgens et al. 2020; Nim et al. 2024), days (Downie et al. 2016) and even within the same day (Costa et al. 2021). Earlier methodological studies suggested that allowing participants to view previous responses may reduce within‐person variability and improve consistency in the use of response scales across repeated assessments, without necessarily attenuating meaningful symptom change (Guyatt et al. 1985). However, these findings have not been evaluated in a large‐scale longitudinal symptom monitoring setting.

During repeated pain monitoring, pain scores are not fully independent assessments because the same individual repeatedly reports on a temporally continuous and fluctuating symptom experience over time (Mueller‐Peltzer et al. 2020). In addition, participants may implicitly or explicitly reference recent symptom experiences or previous responses when rating current pain intensity. Explicitly presenting a participant's previous pain score may therefore influence how individuals calibrate current symptom ratings across repeated assessments and may affect the interpretation of longitudinal pain intensity data in research setting (Kroenke 2018; Lewinson and Katz 2020; Boring et al. 2021; Cernat and Oberski 2023; Yoo et al. 2023). If feedback on previous scores influences the consistency of symptom reporting, this may alter observed within‐person variability during long‐term symptom monitoring. However, the extent to which explicitly presenting prior pain ratings influences within‐person variability in the context of repeated weekly pain monitoring remains unclear (Karimi et al. 2016; Costa et al. 2019, 2021; Butt et al. 2022).

Therefore, the aim of this study was to investigate whether explicit prior‐score feedback influenced within‐person variability in weekly pain intensity ratings during 60 weeks of symptom monitoring in people with spinal pain. A secondary aim was to examine whether prior‐score feedback influenced participant retention during follow‐up. We hypothesised that participants receiving explicit prior‐score feedback would demonstrate lower within‐person variability in weekly pain intensity ratings compared with participants who did not receive prior‐score feedback.

## 2 Methods

### 2.1 Design and Setting

This randomised study evaluating a methodological feature of repeated pain assessment was embedded within the PATH‐Back cohort, a prospective observational study of adults referred for assessment and management of spinal pain at the Spine Centre of Southern Denmark, an outpatient public hospital department. The cohort prospectively followed participants for 60 consecutive weeks through automated SMSs containing a secure link to a REDCap survey. Within this framework, participants were randomised 1:1 at the individual level to complete weekly pain ratings with feedback on their prior week's score or without such feedback. We employed a superiority framework, hypothesizing that feedback would reduce within‐person variability in weekly pain reports relative to no feedback. All analyses were pre‐specified and conducted under blinded group labels and the protocol and statistical analysis plan was uploaded a priori to Open Science Framework on October 20, 2025 (https://osf.io/ngq29). Reporting followed the Consolidated Standards of Reporting Trials (CONSORT) 2025 guidelines (Hopewell et al. 2025). The PATH‐Back cohort is registered with the Odense Patient data Explorative Network's (OPEN) internal project list on July 11, 2022 (https://open.rsyd.dk/projekter/projektliste/projektdetaljer?project=1748). All participants provided written informed consent to participate and the Regional Committees on Health Research Ethics for Southern Denmark (S‐20222000‐1) determined that a formal ethics application was not required.

### 2.2 Eligibility Criteria and Recruitment

Adults (≥ 18 years) referred to the Spine Centre of Southern Denmark with spinal pain defined as neck, mid‐back and/or low back pain were invited to the PATH‐Back cohort and were thereby eligible for inclusion in the embedded randomised study. As part of routine care, all patients completed a standardised electronic clinical registry, SpineData, prior to their first consultation (Kent et al. 2015). Immediately after completing the registry, patients were consecutively invited to participate in the study. Eligibility additionally required ownership of a smartphone to receive weekly SMS messages with the REDCap survey link and the ability to read Danish. No other exclusion criteria were applied at the cohort level. For analysis of the embedded trial, participants also needed to have responded to at least 40% of the weekly SMS messages (≥ 24 of 60 weeks) to allow estimation of within‐person pain variability over time.

### 2.3 Intervention and Comparator

Each week, participants rated their average spinal pain intensity during the past week on a 0–10 numerical rating scale (NRS) using the question: ‘How intense has your back pain [low back, mid back and/or neck] typically been during the past week, on a scale from 0 to 10?’. Participants were randomised to:

*Intervention (feedback):* Participants received an SMS displaying their previous week's score within the message. The wording was: ‘You answered [X] last week’, where [X] was the prior week's NRS value. If no response had been recorded the previous week, the feedback field remained blank.

*Comparator (no feedback):* Participants received no references to their previous score.

Apart from the feedback element, all procedures were identical across groups.

### 2.4 Sample Size

The PATH‐Back cohort was initially designed to recruit approximately 2000 participants to support longitudinal analyses of pain trajectories and related outcomes. Due to low retention of ~1/3, this was increased to 2500 participants during the inclusion period. Although the cohort was not specifically powered for the present embedded methodological analyses, the large sample size and repeated weekly assessments provided substantial longitudinal data for estimation and comparison of within‐person variability across groups.

### 2.5 Randomization, Allocation and Blinding

#### 2.5.1 Sequence Generation

Participants were randomised in a 1:1 ratio to the feedback or no‐feedback arm using computer‐generated simple randomisation at baseline. The randomisation algorithm was implemented centrally within REDCap by an independent data manager at OPEN.

#### 2.5.2 Allocation Concealment

The allocation sequence was embedded within the REDCap survey logic. Because assignment was stored and implemented server‐side, it was not visible to participants or researchers during data collection. The OPEN data manager created the branching logic controlling the intervention, ensuring complete allocation concealment.

#### 2.5.3 Blinding

Participants could infer their group assignment if the prior score was displayed, so full blinding was not feasible. However, they were unaware that the study aimed to evaluate feedback effects; all participants were informed only that they would receive weekly SMS questions. Investigators remained blinded throughout all analyses. An independent data manager provided a de‐identified dataset containing an A/B group variable, where one label corresponded to the feedback arm and the other to control. All analyses were conducted on the A/B‐blinded dataset. The data manager disclosed the A/B mapping only after the full manuscript was written and all inferences were drawn.

### 2.6 Adverse Events

Because this was a methodological study with no clinical intervention, no harms or adverse events were expected or actively monitored. Participants who contacted the study team to withdraw from SMS follow‐up were asked to provide a reason, including any potential adverse experiences.

### 2.7 Baseline Characteristics Variables

The following characteristics were extracted from SpineData:

- Demographics: sex (male/female), age (years), body mass index (BMI, kg/m^2^) and highest completed education (basic, vocational or higher).
- Work‐related: self‐reported sick leave within the last 3 months due to back pain (yes/no).
- Clinical characteristics: Spinal pain region (low back or neck/mid‐back), duration (< 3 months, 3–12 months, 12–24 months or ≥ 24 months), intensity (0–10 numerical rating scale) (Manniche et al. 1994); extremity pain intensity (0–10 numerical rating scale), disability measured using the Oswestry Disability Index (ODI, 0%–100%) or the Neck Disability Index (NDI, 0%–100%) according to spinal pain region (Fairbank and Pynsent 2000; Vernon 2008) and the STarT Back Tool classification (low, medium or high risk) (Hill et al. 2008).

### 2.8 Outcome Variables

#### 2.8.1 Primary Outcome

The primary outcome was within‐person variability in weekly pain intensity across the 60‐week follow‐up, operationalised as the *root mean square successive differences* (RMSSD). RMSSD quantifies short‐term fluctuations by calculating the average magnitude of successive week‐to‐week changes in pain intensity (Berntson et al. 1997). RMSSD was selected as the primary outcome because it is a well‐established index of short‐term variability in time‐series data and provides a single summary measure that can be directly compared across groups (30, 31). Full rationale and computation details are available in the preregistered protocol and statistical analysis plan (https://osf.io/ngq29).

#### 2.8.2 Secondary Outcomes

Additional fluctuation metrics derived from weekly pain ratings:

- Standard deviation (SD): overall dispersion of pain scores around each participant's mean.
- Mean absolute successive differences (MASD): average absolute week‐to‐week change.
- Root mean square error (RMSE): deviation of weekly pain scores from each participant's fitted linear trend.
- Proportion of absolute change (PAC1 and PAC2): proportion of weeks with ≥ 1‐point and ≥ 2‐point deviations from the participant's mean, respectively.

#### 2.8.3 Exploratory Secondary Outcome

The proportion of missing weekly SMS responses was examined as an indicator of reporting completeness.

### 2.9 Statistical Analysis

All analyses followed a modified intention‐to‐treat principle by including all randomised participants who provided at least 40% of the weekly SMS responses (≥ 24 of 60 weeks). No imputations were performed.

#### 2.9.1 Descriptive Statistics

A CONSORT flow diagram was conducted. Baseline characteristics were summarised stratified by groups. Continuous variables were presented as means with standard deviations (SD) and categorical variables as counts and percentages. For the SMS‐based follow‐up, adherence was summarised as the mean (SD, range) number of weekly replies per participant.

#### 2.9.2 Comparative Analyses

For each participant, RMSSD was calculated across all available weekly pain ratings during the 60‐week follow‐up period. Mean RMSSD values were compared between the feedback and no‐feedback groups using linear regression, with RMSSD as the dependent variable and group allocation as the independent variable. Secondary outcomes (SD, MASD, RMSE, PAC1 and PAC2) were analysed using the same modelling approach. Given the exploratory nature of these outcomes, no adjustment for multiple comparisons was applied. Results were expressed as mean differences with corresponding 95% confidence intervals. SMS retention was analysed by modelling the number of weeks with missing SMS responses (0–36) using Poisson regression. Model assumptions were assessed using diagnostic plots; for the linear regression models, residual normality was evaluated using QQ plots, heteroskedasticity using plots of residuals against fitted values and influential observations using standardised residuals and influence diagnostics. For the Poisson model, overdispersion was assessed using the ratio of the Pearson chi‐square statistic to the residual degrees of freedom. Finally, descriptive analyses were used to visualise the distributions of pain variability and SMS retention using violin plots with embedded boxplots and individual observations.

#### 2.9.3 Sensitivity Analyses

All analyses were repeated using a stricter inclusion threshold of ≥ 60% valid weekly SMS responses (≥ 36/60) and as a complete‐case analysis. To examine robustness to possible baseline imbalances, all regression models were repeated with covariate adjustment using the pre‐specified baseline variables (see above). To test whether the assumed linear trend influenced estimation of residual variability, RMSE analysis was repeated using quadratic and cubic individual trend models.

The statistical analysis was conducted in R v. 4.5 (R Development Core Team 2009). Data wrangling, synthesis and visualisations were performed using the Tidyverse language (Wickham et al. 2019). Model checks were conducted using *check_model*() from the performance package (Makowski et al. 2020).

## 3 Results

**FIGURE 1:**
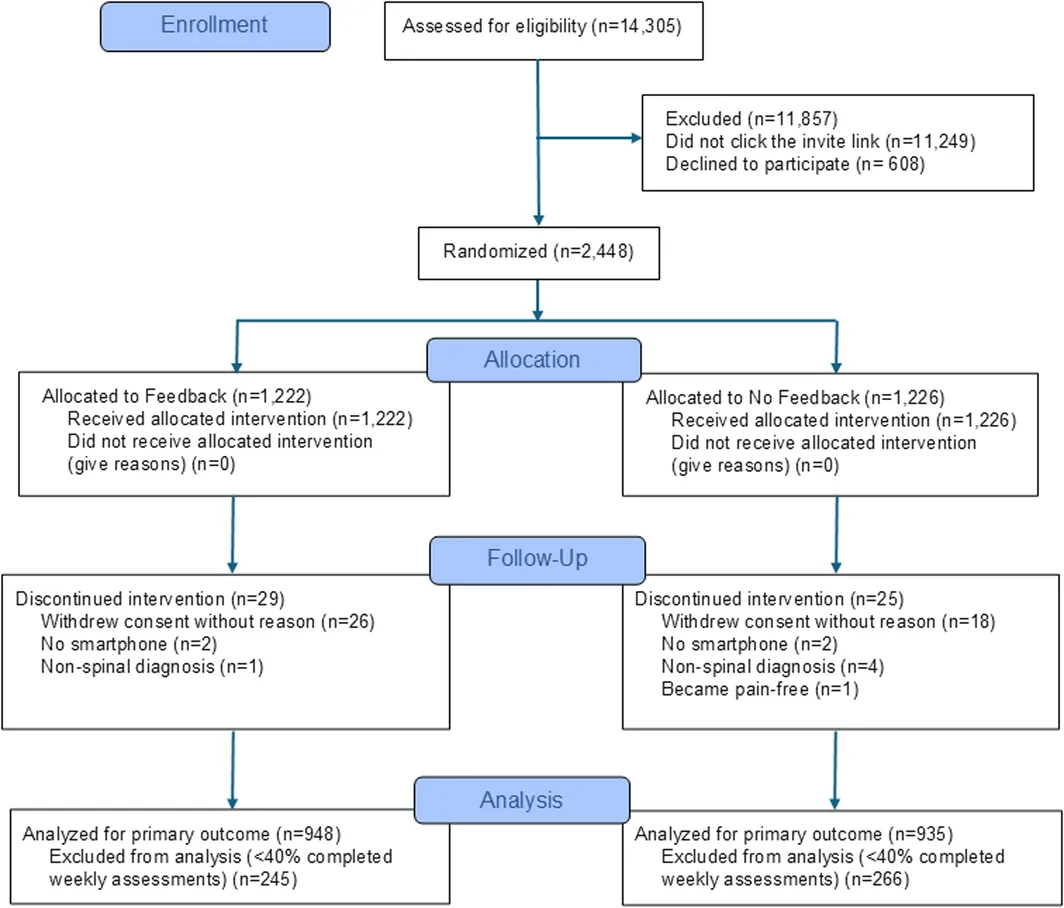
CONSORT flowchart.

A total of 2448 participants were enrolled between February 2, 2023 and May 19, 2024, with the final follow‐up at July 6, 2025. Each participant was randomised to the feedback group (*n* = 1222) or the no‐feedback group (*n* = 1226). Corresponding to 40% valid weekly responses, 1883 participants (77%) were included in the analyses (Figure 1). Baseline characteristics were comparable between groups (Supporting Information S1.1). The cohort primarily consisted of participants with low back pain (86%), 54% women and mean age 60 years (SD 13). Mean disability was 35 points (SD 15) on a 0–100 scale for both ODI and NDI. Mean spinal and extremity pain intensities were 6/10 and 5/10, respectively and 44% were classified as high‐risk. On average, participants replied to 93% (SD 14) of weekly SMS messages, with no differences between groups (Supporting Information S1.1). Participants excluded due to < 40% response rate were on average younger (mean 55 years) and more likely to have been on sick leave than those included (Supporting Information S2).

**FIGURE 2:**
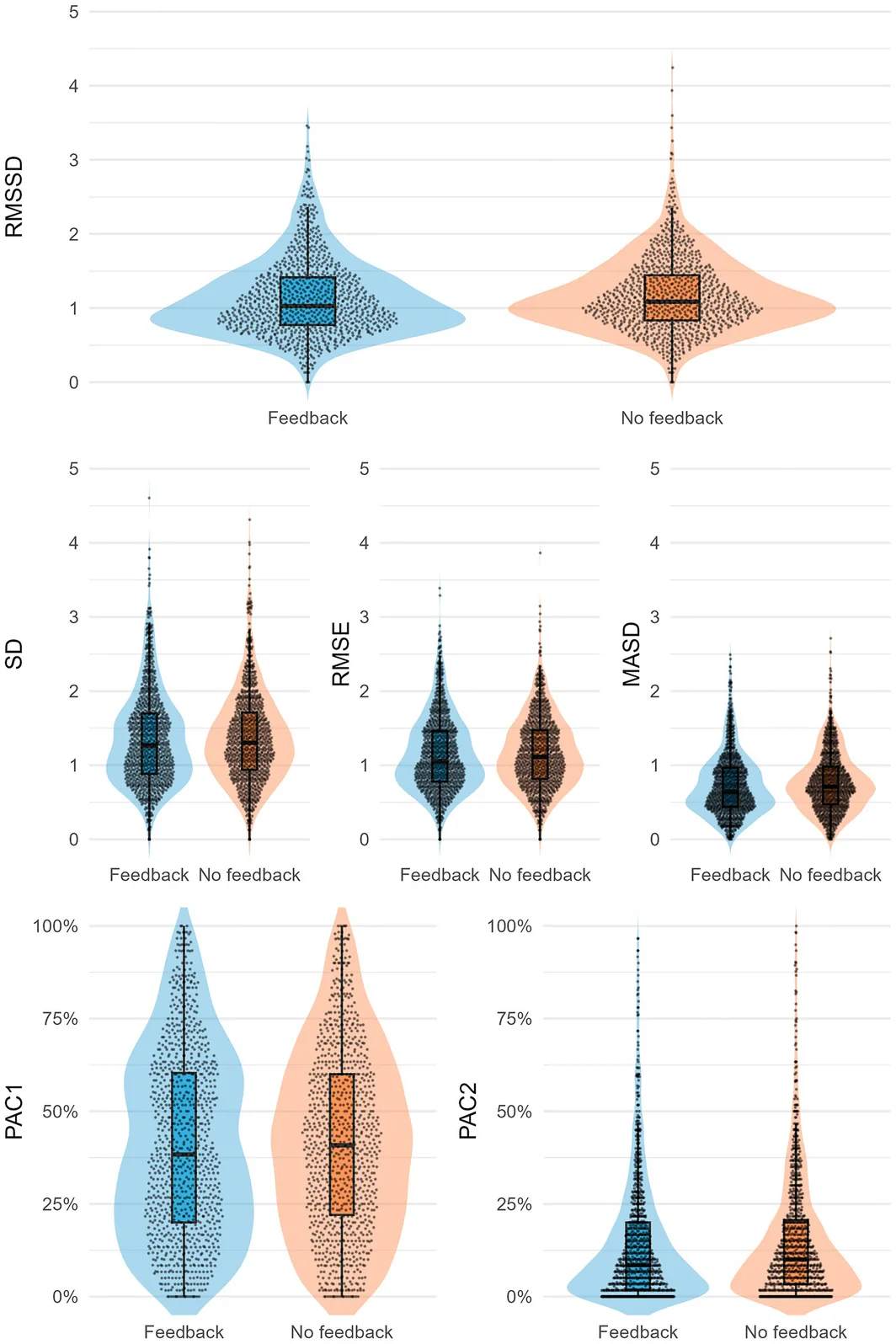
Descriptive within‐individual variability measures. MASD, mean absolute successive difference; PAC1, proportion of weeks with ≥ 1‐point change; PAC2, proportion of weeks with ≥ 2‐point change; RMSE, root mean square error; RMSSD, root mean square of successive differences; SD, standard deviation.

**FIGURE 3:**
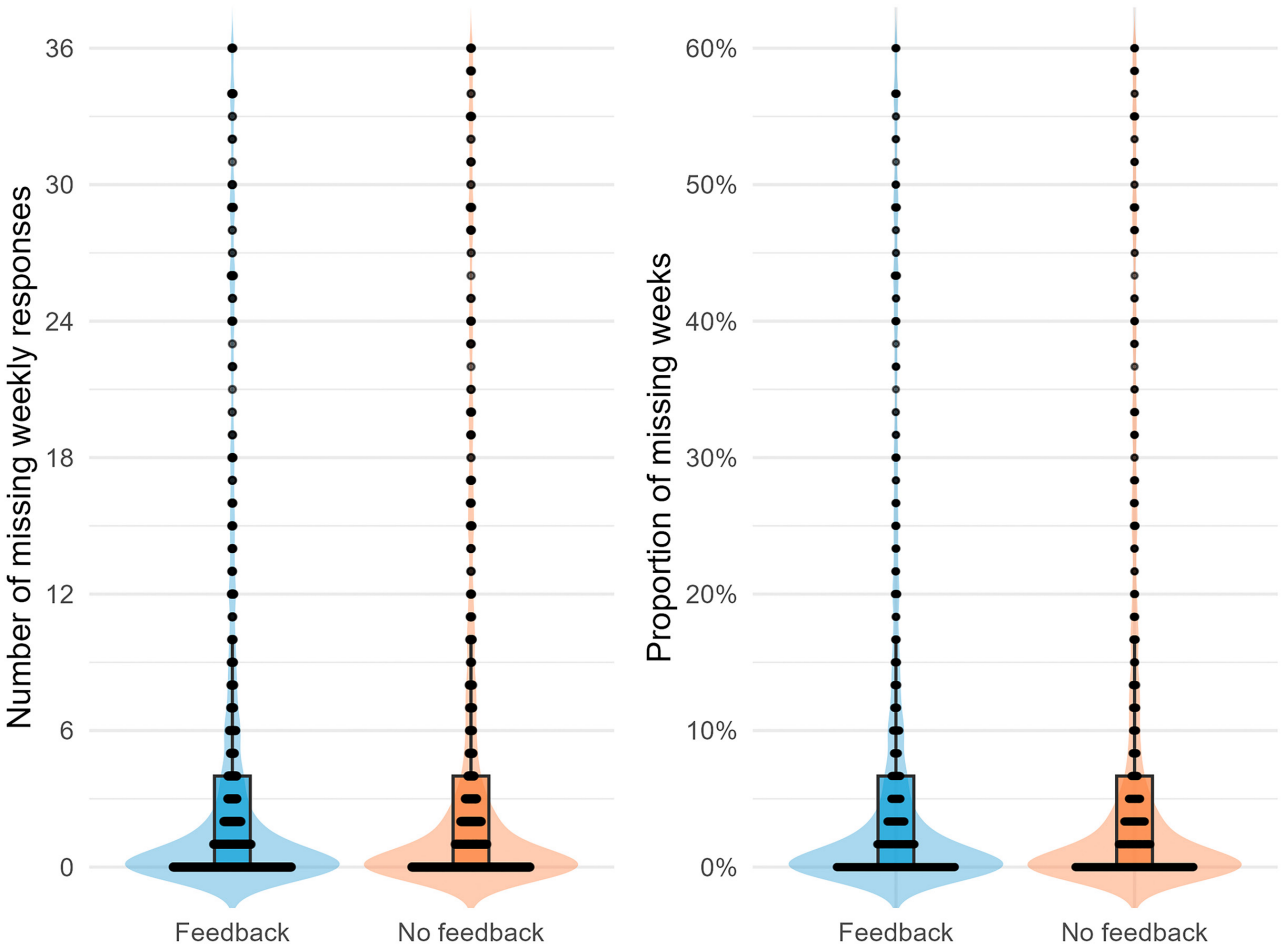
Number of weeks with missing responses.

Model diagnostics for RMSSD, SD, RMSE and MASD supported adequate model fit and for PAC1 and PAC2, minor deviations from normality were shown (Supporting Information S3, Figure 2). However, for SMS retention the data were clearly overdispersed and a negative binomial model with a log link was used to obtain rate ratios with 95% confidence intervals (Supporting Information S3, Figure 3).

No between‐group differences were observed for the unadjusted primary or secondary outcomes. After covariate adjustment, small differences were observed for RMSSD (−0.05 [−0.10, −0.00]) and Mean Absolute Successive Differences (−0.04 [−0.08, −0.01]), whereas all other outcomes remained comparable between groups. Likewise, the proportion of missing SMS responses did not differ between groups (Table 1). In the complete‐case analysis, the adjusted group difference was slightly larger than in the main analysis. All other sensitivity analyses yielded consistent results (Supporting Information S4).

**TABLE 1:** Between group differences for within‐individual variability measures and weeks with missing SMS responses.

Outcome	Model	Mean difference (95% CI)	*p*
Root mean square of successive differences	Unadjusted	−0.039 (−0.086, 0.008)	0.103
	Adjusted	−0.054 (−0.103, −0.004)	0.034
Standard deviation of weekly pain intensity	Unadjusted	−0.018 (−0.075, 0.040)	0.546
	Adjusted	−0.030 (−0.090, 0.031)	0.336
Mean absolute successive differences	Unadjusted	−0.034 (−0.072, 0.004)	0.078
	Adjusted	−0.044 (−0.084, −0.005)	0.028
Root mean square error of residuals	Unadjusted	−0.014 (−0.059, 0.032)	0.555
	Adjusted	−0.023 (−0.072, 0.026)	0.353
Proportion of weeks with ≥ 1‐point change	Unadjusted	−0.009 (−0.032, 0.013)	0.418
	Adjusted	−0.011 (−0.035, 0.013)	0.386
Proportion of weeks with ≥ 2‐point change	Unadjusted	−0.007 (−0.023, 0.009)	0.401
	Adjusted	−0.011 (−0.028, 0.006)	0.207
Weeks with missing data	Unadjusted	0.972 (0.806, 1.172)	0.764
	Adjusted	0.950 (0.776, 1.162)	0.612

*Note:* All estimates represent mean differences, except for ‘Weeks with missing data’, which reports rate ratios from a negative binomial model. Adjusted models include sex, age, body mass index, education, sick leave within the last 3 months due to back pain, primary pain location, pain duration, local spinal pain, extremity pain, *Z*‐scaled Oswestry Disability Index or the Neck Disability Index and STarT Back Tool classification.

Across all variability measures, there was considerable within‐individual variation in weekly pain scores, with median RMSSD values for NRS ratings typically ranging between 1 and 2 points (Figure 2). The distributions were approximately normal, with few participants showing either no variability or extreme fluctuations. PAC1 events were relatively frequent (proportion of weeks with ≥ 1‐point deviations from the participant's mean), whereas PAC2 events (≥ 2‐points) were rare (median ~10%). Likewise, the median number of weeks with missing SMS responses was close to zero, although a small number of participants had missing data approaching the inclusion threshold (Figure 3).

## 4 Discussion

This study evaluated whether adding information on prior pain scores to a weekly SMS‐based pain‐monitoring system affected within‐person variability or retention. Despite substantial within‐person fluctuations in weekly pain intensity, we observed no meaningful between‐group differences in any variability measures. The feedback also did not affect SMS response rates (missingness), indicating that such feedback does not systematically impact longitudinal pain self‐reports.

We used a randomised design with blinded, preregistered analyses and results were nearly identical across all sensitivity checks. In the complete‐case analysis, the adjusted group difference was slightly larger, which may reflect that participants with more complete follow‐up (i.e., more repeated exposure to the feedback) displayed marginally lower variability. However, the absolute magnitude of this difference remained small and arguably not clinically meaningful.

Research examining whether feedback of patient‐reported outcome measures influences variability has been limited. Early work suggested that access to prior responses may reduce variability over very short recall periods when no clinical change is expected (Guyatt et al. 1985). In contrast, consistent with our findings, studies using longer recall intervals or conducted in settings where clinical change was anticipated have generally found little to no effect of feedback on the consistency of symptom reporting across repeated assessments (Guyatt et al. 1989; Schünemann et al. 2002; Silverman et al. 2011).

Feedback on prior patient‐reported outcomes may theoretically influence how individuals interpret, recall or calibrate symptoms across repeated assessments (Sprangers et al. 1999). Reporting mechanisms, such as reactivity bias (French and Sutton 2010) or anchoring to previous responses (Furnham and Boo 2011), could potentially influence repeated pain reports over time. In the context of weekly pain monitoring, such processes might be expected to produce systematic differences in variability or reporting patterns between groups (Wittich et al. 2024). However, despite prolonged exposure to prior‐score feedback over 60 weeks, we observed minimal between‐group differences across all variability metrics. Although these processes were not directly measured in the present study, the findings suggest that explicit prior‐score feedback had limited influence on repeated pain reporting under these monitoring conditions.

Repeated longitudinal pain assessments have revealed substantial within‐person variation and heterogeneous pain courses, motivating trajectory‐ and variability‐focused analyses (Irgens et al. 2020; Kongsted et al. 2016, 2017; Nim et al. 2024). In this randomised comparison, the magnitude of this variation was largely unaffected by whether participants were shown their previous pain score. Although these findings do not validate longitudinal modelling approaches (Kongsted and Nielsen 2017; van de Schoot et al. 2017), they provide specific methodological reassurance that the observed variation is robust to this aspect of study design.

Our findings also demonstrated substantial week‐to‐week fluctuations in pain intensity among individuals with spinal pain receiving specialised care, consistent with previous longitudinal studies in spinal pain populations (Andersen et al. 2022; Dutmer et al. 2020). Importantly, these fluctuations remained largely unchanged despite prolonged exposure to prior‐score feedback over 60 weeks. This supports the use of repeated pain measurements and variability‐focused metrics, such as RMSSD and PAC, as complementary approaches to mean pain intensity and trajectory‐based modelling (Kongsted and Nielsen 2017; Nim et al. 2023; van de Schoot et al. 2017). Future research should examine whether within‐person variability affects outcomes such as sick leave, work participation or disability and whether incorporating variability metrics can enhance modelling and interpretation of pain dynamics. Additionally, time‐series–based approaches may provide further insight into how short‐term variability can be used to estimate when symptoms stabilise or ‘settle’ (Mueller‐Peltzer et al. 2020).

Some limitations should be acknowledged. First, participants were recruited from secondary care and represent individuals with persistent and often complex pain presentations. Thus, our findings may not generalise to populations with more recent symptom onset, where week‐to‐week pain intensity can change considerably or where there is a greater likelihood of spontaneous recovery. Second, only 17% of the eligible population responded to the initial invitation. Although this response rate introduces potential selection bias, the resulting sample remains large for a longitudinal pain‐monitoring study. We have no reason to suspect that feedback would affect non‐responders differently. Third, excluding participants with fewer than 40% valid weekly responses may have biased the sample toward older responders. Finally, in our analysis for PAC1 and PAC2, we found some deviations from normality; this was expected due to the bound nature and zero‐inflation of these proportion measures, particularly for PAC2, where many participants showed no large fluctuations. These deviations introduced a distributional mismatch that was unlikely to affect the robustness of the between‐group comparisons and we completed our pre‐planned analysis.

## 5 Conclusion

Providing feedback about the previous week's pain score did not meaningfully alter short‐term pain variability or retention during 60 weeks of weekly monitoring. Substantial within‐person variability in pain intensity was observed across follow‐up and this variability was largely unaffected by explicit prior‐score feedback. These findings contribute to the interpretation of repeated longitudinal pain assessments in spinal pain research and suggest that weekly pain reporting patterns are robust to this feedback during long‐term symptom monitoring.

## Author Contributions

Casper Nim, Alice Kongsted: conceptualization; Casper Nim, Søren O'Neill: data curation; Casper Nim: investigation, project administration, writing – original draft; Casper Nim, Aron Downie, Alice Kongsted: methodology; Casper Nim, Aron Downie, Søren O'Neill, Rikke Krüger Jensen, Sheilah Hogg‐Johnson, Alice Kongsted: writing – review and editing.

## Funding

This study was financially supported by the Danish Foundation for Chiropractic Research and Post‐graduate Education (grant A4839) and Hospital Lillebaelt (internal grant). CN was supported by a clinical‐research career grant from the Region of Southern Denmark (grant 21/67684). The funders had no role in study design, data collection, analysis, interpretation or report writing.

## Conflicts of Interest

The authors declare no conflicts of interest.

## Supporting information


**Table S1:1.** Baseline characteristics.
**Table S1:2:** SMS summary.
**Table S2:1:** Retention analysis.
**Figure S3:1:** Model check root mean square of successive differences.
**Figure S3:2:** Model check standard deviation.
**Figure S3:3:** Model check mean absolute successive differences.
**Figure S3:4:** Model check root mean square error of residuals.
**Figure S3:5:** Model check proportion of Weeks with ≥ 1‐point change.
**Figure S3:6:** Model check proportion of Weeks with ≥ 2‐point change.
**Table S4:1:** Group differences for RSME with higher orders time trends.
**Table S4:2:** Regression results—sensitivity for inclusion.

## References

[ejp70361-bib-0001] Andersen, T. E. , K.‐I. Karstoft , H. H. Lauridsen , and C. Manniche . 2022. “Trajectories of Disability in Low Back Pain.” Pain Reports 7: e985. 35047714 10.1097/PR9.0000000000000985 PMC8765574

[ejp70361-bib-0002] Berntson, G. G. , J. T. Bigger , D. L. Eckberg , et al. 1997. “Heart Rate Variability: Origins, Methods, and Interpretive Caveats.” Psychophysiology 34: 623–648. 9401419 10.1111/j.1469-8986.1997.tb02140.x

[ejp70361-bib-0003] Boring, B. L. , K. T. Walsh , N. Nanavaty , B. W. Ng , and V. A. Mathur . 2021. “How and Why Patient Concerns Influence Pain Reporting: A Qualitative Analysis of Personal Accounts and Perceptions of Others' Use of Numerical Pain Scales.” Frontiers in Psychology 12: 663890. 34282355 10.3389/fpsyg.2021.663890 PMC8285731

[ejp70361-bib-0004] Butt, B. B. , D. Kagan , J. Gagnier , et al. 2022. “Do Patients Accurately Recall Their Pain Levels Following Epidural Steroid Injection? A Cohort Study of Recall Bias in Patient‐Reported Outcomes.” Pain Physician 25: 59–66. 35051145

[ejp70361-bib-0005] Cernat, A. , and D. Oberski . 2023. “Estimating Measurement Error in Longitudinal Data Using the Longitudinal MultiTrait MultiError Approach.” Structural Equation Modeling: A Multidisciplinary Journal 30: 592–603.

[ejp70361-bib-0006] Costa, N. , M. L. Ferreira , J. Setchell , et al. 2019. “A Definition of ‘Flare’ in Low Back Pain: A Multiphase Process Involving Perspectives of Individuals With Low Back Pain and Expert Consensus.” Journal of Pain 20: 1267–1275. 30904517 10.1016/j.jpain.2019.03.009

[ejp70361-bib-0007] Costa, N. , E. J. Smits , J. Kasza , S. E. Salomoni , M. Ferreira , and P. W. Hodges . 2021. “Low Back Pain Flares: How Do They Differ From an Increase in Pain?” Clinical Journal of Pain 37: 313–320. 33830090 10.1097/AJP.0000000000000926

[ejp70361-bib-0008] Downie, A. S. , M. J. Hancock , M. Rzewuska , C. M. Williams , C.‐W. C. Lin , and C. G. Maher . 2016. “Trajectories of Acute Low Back Pain: A Latent Class Growth Analysis.” Pain 157: 225–234. 26397929 10.1097/j.pain.0000000000000351

[ejp70361-bib-0009] Dunn, K. M. , P. Campbell , and K. P. Jordan . 2013. “Long‐Term Trajectories of Back Pain: Cohort Study With 7‐Year Follow‐Up.” BMJ Open 3: e003838. 10.1136/bmjopen-2013-003838 PMC3863121 24334157

[ejp70361-bib-0010] Dunn, K. M. , P. Campbell , and K. P. Jordan . 2017. “Validity of the Visual Trajectories Questionnaire for Pain.” Journal of Pain 18: 1451–1458. 28842368 10.1016/j.jpain.2017.07.011 PMC5722486

[ejp70361-bib-0011] Dutmer, A. L. , H. R. Schiphorst Preuper , R. E. Stewart , R. Soer , M. F. Reneman , and A. P. Wolff . 2020. “Trajectories of Disability and Low Back Pain Impact.” Spine 45: 1649–1660. 32833933 10.1097/BRS.0000000000003647 PMC7647438

[ejp70361-bib-0012] Fairbank, J. C. , and P. B. Pynsent . 2000. “The Oswestry Disability Index.” Spine 25: 2940–2952; discussion 2952. 11074683 10.1097/00007632-200011150-00017

[ejp70361-bib-0013] Ferreira, M. L. , K. de Luca , L. M. Haile , et al. 2023. “Global, Regional, and National Burden of Low Back Pain, 1990–2020, Its Attributable Risk Factors, and Projections to 2050: A Systematic Analysis of the Global Burden of Disease Study 2021.” Lancet Rheumatology 5: e316–e329. 37273833 10.1016/S2665-9913(23)00098-X PMC10234592

[ejp70361-bib-0014] French, D. P. , and S. Sutton . 2010. “Reactivity of Measurement in Health Psychology: How Much of a Problem Is It? What Can Be Done About It?” British Journal of Health Psychology 15: 453–468. 20205982 10.1348/135910710X492341

[ejp70361-bib-0015] Furnham, A. , and H. C. Boo . 2011. “A Literature Review of the Anchoring Effect.” Journal of Socio‐Economics 40: 35–42.

[ejp70361-bib-0016] Guyatt, G. H. , L. B. Berman , M. Townsend , and D. W. Taylor . 1985. “Should Study Subjects See Their Previous Responses?” Journal of Chronic Diseases 38: 1003–1007. 4066888 10.1016/0021-9681(85)90098-0

[ejp70361-bib-0017] Guyatt, G. H. , M. Townsend , J. L. Keller , and J. Singer . 1989. “Should Study Subjects See Their Previous Responses: Data From a Randomized Control Trial.” Journal of Clinical Epidemiology 42: 913–920. 2778469 10.1016/0895-4356(89)90105-4

[ejp70361-bib-0018] Hartvigsen, J. , M. J. Hancock , A. Kongsted , et al. 2018. “What Low Back Pain Is and Why We Need to Pay Attention.” Lancet 391: 2356–2367. 29573870 10.1016/S0140-6736(18)30480-X

[ejp70361-bib-0019] Hill, J. C. , K. M. Dunn , M. Lewis , et al. 2008. “A Primary Care Back Pain Screening Tool: Identifying Patient Subgroups for Initial Treatment.” Arthritis and Rheumatism 59: 632–641. 18438893 10.1002/art.23563

[ejp70361-bib-0020] Hopewell, S. , A.‐W. Chan , G. S. Collins , et al. 2025. “CONSORT 2025 Statement: Updated Guideline for Reporting Randomised Trials.” BMJ: e081123. 40228833 10.1136/bmj-2024-081123 PMC11995449

[ejp70361-bib-0021] Irgens, P. , A. Kongsted , B. L. Myhrvold , et al. 2020. “Neck Pain Patterns and Subgrouping Based on Weekly SMS‐Derived Trajectories.” BMC Musculoskeletal Disorders 21: 678. 33054732 10.1186/s12891-020-03660-0 PMC7559200

[ejp70361-bib-0022] Karimi, Z. , A. Pilenko , S. M. Held , and M. I. Hasenbring . 2016. “Recall Bias in Patients With Chronic Low Back Pain: Individual Pain Response Patterns Are More Important Than Pain Itself!” International Journal of Behavioral Medicine 23: 12–20. 26154771 10.1007/s12529-015-9499-6

[ejp70361-bib-0023] Kent, P. , A. Kongsted , T. S. Jensen , H. B. Albert , B. Schiøttz‐Christensen , and C. Manniche . 2015. “SpineData—a Danish Clinical Registry of People With Chronic Back Pain.” Clinical Epidemiology 7: 369–380. 26316820 10.2147/CLEP.S83830 PMC4540159

[ejp70361-bib-0024] Kongsted, A. , L. Hestbæk , and P. Kent . 2017. “How Can Latent Trajectories of Back Pain Be Translated Into Defined Subgroups.” BMC Musculoskeletal Disorders 18: 285. 28673341 10.1186/s12891-017-1644-8 PMC5496263

[ejp70361-bib-0025] Kongsted, A. , T. S. Jensen , K. Doktor , and L. Hestbæk . 2021. “Effects of Weekly Pain Monitoring on Back Pain Outcomes: A Non‐Randomised Controlled Study.” Chiropractic & Manual Therapies 29: 37. 34530882 10.1186/s12998-021-00393-2 PMC8444569

[ejp70361-bib-0026] Kongsted, A. , P. Kent , I. Axen , A. S. Downie , and K. M. Dunn . 2016. “What Have We Learned From Ten Years of Trajectory Research in Low Back Pain?” BMC Musculoskeletal Disorders 17: 220. 27209166 10.1186/s12891-016-1071-2 PMC4875630

[ejp70361-bib-0027] Kongsted, A. , and A. M. Nielsen . 2017. “Latent Class Analysis in Health Research.” Journal of Physiotherapy 63: 55–58. 27914733 10.1016/j.jphys.2016.05.018

[ejp70361-bib-0028] Kroenke, K. 2018. “Pain Measurement in Research and Practice.” Journal of General Internal Medicine 33: 7–8. 10.1007/s11606-018-4363-4 PMC5902352 29633135

[ejp70361-bib-0029] Lewinson, R. E. , and J. D. Katz . 2020. “Influencing Pain Inferences Using Random Numerical Anchoring: Randomized Controlled Trial.” JMIR Human Factors 7: e17533. 32149719 10.2196/17533 PMC7091028

[ejp70361-bib-0030] Makowski, D. , M. S. Ben‐Shachar , and D. Lüdecke . 2020. The Easystats Collection of R Packages. GitHub.

[ejp70361-bib-0031] Manniche, C. , K. Asmussen , B. Lauritsen , H. Vinterberg , S. Kreiner , and A. Jordan . 1994. “Low Back Pain Rating Scale: Validation of a Tool for Assessment of Low Back Pain.” Pain 57: 317–326. 7936710 10.1016/0304-3959(94)90007-8

[ejp70361-bib-0032] Mueller‐Peltzer, M. , S. Feuerriegel , A. Molgaard Nielsen , A. Kongsted , W. Vach , and D. Neumann . 2020. “Longitudinal Healthcare Analytics for Disease Management: Empirical Demonstration for Low Back Pain.” Decision Support Systems 132: 113271.

[ejp70361-bib-0033] Nim, C. , A. S. Downie , A. Kongsted , et al. 2024. “Prospective Back Pain Trajectories or Retrospective Recall—Which Tells Us Most About the Patient?” Journal of Pain 25: 104555. 38719157 10.1016/j.jpain.2024.104555

[ejp70361-bib-0034] Nim, C. G. , W. Vach , A. Downie , and A. Kongsted . 2023. “Do Visual Pain Trajectories Reflect the Actual Course of Low Back Pain? A Longitudinal Cohort Study.” Journal of Pain 24: 1506–1521. 37044294 10.1016/j.jpain.2023.04.004

[ejp70361-bib-0035] R Development Core Team . 2009. R: A Language and Environment for Statistical Computing. R Foundation for Statistical Computing.

[ejp70361-bib-0036] Schünemann, H. J. , G. H. Guyatt , L. Griffith , D. Stubbing , and R. Goldstein . 2002. “A Randomized Controlled Trial to Evaluate the Effect of Informing Patients About Their Pretreatment Responses to Two Respiratory Questionnaires.” Chest 122: 1701–1708. 12426274 10.1378/chest.122.5.1701

[ejp70361-bib-0037] Silverman, S. , M. Cates , and G. Saunders . 2011. “Is Measured Hearing Aid Benefit Affected by Seeing Baseline Outcome Questionnaire Responses?” American Journal of Audiology 20: 90–99. 21940983 10.1044/1059-0889(2011/10-0003) PMC4724418

[ejp70361-bib-0038] Sprangers, M. A. , F. S. van Dam , J. Broersen , et al. 1999. “Revealing Response Shift in Longitudinal Research on Fatigue–the Use of the Thentest Approach.” Acta Oncologica 38: 709–718. 10522761

[ejp70361-bib-0039] van de Schoot, R. , M. Sijbrandij , S. D. Winter , S. Depaoli , and J. K. Vermunt . 2017. “The GRoLTS‐Checklist: Guidelines for Reporting on Latent Trajectory Studies.” Structural Equation Modeling: A Multidisciplinary Journal 24: 451–467.

[ejp70361-bib-0040] Vernon, H. 2008. “The Neck Disability Index: State‐Of‐The‐Art, 1991–2008.” Journal of Manipulative and Physiological Therapeutics 31: 491–502. 18803999 10.1016/j.jmpt.2008.08.006

[ejp70361-bib-0041] Wickham, H. , M. Averick , J. Bryan , et al. 2019. “Welcome to the Tidyverse.” Journal of Open Source Software 4: 1686.

[ejp70361-bib-0042] Wittich, L. , C. Tsatsaronis , D. Kuklinski , et al. 2024. “Patient‐Reported Outcome Measures as an Intervention: A Comprehensive Overview of Systematic Reviews on the Effects of Feedback.” Value in Health 27: 1436–1453. 38843978 10.1016/j.jval.2024.05.013

[ejp70361-bib-0043] Wu, A.‐M. , M. Cross , J. M. Elliott , et al. 2024. “Global, Regional, and National Burden of Neck Pain, 1990–2020, and Projections to 2050: A Systematic Analysis of the Global Burden of Disease Study 2021.” Lancet Rheumatology 6: e142–e155. 38383088 10.1016/S2665-9913(23)00321-1 PMC10897950

[ejp70361-bib-0044] Yoo, H. , Y. Cho , and S. Cho . 2023. “Does Past/Current Pain Change Pain Experience? Comparing Self‐Reports and Pupillary Responses.” Frontiers in Psychology 14: 1094903. 36874838 10.3389/fpsyg.2023.1094903 PMC9982106

