# Supplementary material for: Providing Feedback on Previous Pain Scores Did Not Affect Weekly Pain Variability: A Cohort‐Nested Randomised Study
Source: Eur J Pain. 2026 Aug 20;30(8):e70361. doi: 10.1002/ejp.70361 (PMC13491302; doi:10.1002/ejp.70361)
Supplement: Supplementary file 1 [file EJP-30-0-s001.docx]

# Supplementary files

## Supplementary file 1:

#### Table S1.1: Baseline characteristics

| **Baseline characteristic** | **N** | **Overall**  N = 1,883^1^ | **Feedback**  N = 948^1^ | **No feedback**  N = 935^1^ |  |
| --- | --- | --- | --- | --- | --- |
| **Pain region** | 1,873 |  |  |  |  |
| Low back pain |  | 1,616 (86%) | 811 (86%) | 805 (87%) |  |
| Neck/mid-back pain |  | 257 (14%) | 132 (14%) | 125 (13%) |  |
| Missing |  | 10 | 5 | 5 |  |
| **Sex** | 1,883 |  |  |  |  |
| Female |  | 1,019 (54%) | 515 (54%) | 504 (54%) |  |
| Male |  | 864 (46%) | 433 (46%) | 431 (46%) |  |
| **Age (years)** | 1,883 | 60 (13) | 60 (13) | 61 (13) |  |
| **Body mass index (kg/m²)** | 1,863 | 28.2 (5.6) | 28.4 (5.7) | 28.0 (5.4) |  |
| Missing |  | 20 | 11 | 9 |  |
| **Education level** | 1,750 |  |  |  |  |
| Basic |  | 553 (32%) | 276 (31%) | 277 (32%) |  |
| Middle |  | 954 (55%) | 485 (55%) | 469 (54%) |  |
| Higher |  | 243 (14%) | 120 (14%) | 123 (14%) |  |
| Missing |  | 133 | 67 | 66 |  |
| **Sick leave past 3 months** | 1,734 |  |  |  |  |
| Yes |  | 447 (26%) | 237 (27%) | 210 (24%) |  |
| No |  | 572 (33%) | 278 (32%) | 294 (34%) |  |
| Not assessed |  | 715 (41%) | 353 (41%) | 362 (42%) |  |
| Missing |  | 149 | 80 | 69 |  |
| **Regional pain (0–10)** | 1,873 | 6 (2) | 6 (2) | 6 (2) |  |
| Missing |  | 10 | 4 | 6 |  |
| **Extremity pain (0–10)** | 1,873 | 5 (3) | 5 (3) | 5 (3) |  |
| Missing |  | 10 | 4 | 6 |  |
| **Disability (Z-score)** | 1,872 | -0.01 (0.98) | -0.01 (0.98) | 0.00 (0.98) |  |
| Missing |  | 11 | 5 | 6 |  |
| **STarT Back risk group** | 1,871 |  |  |  |  |
| High risk |  | 815 (44%) | 405 (43%) | 410 (44%) |  |
| Medium risk |  | 509 (27%) | 256 (27%) | 253 (27%) |  |
| Low risk |  | 290 (15%) | 149 (16%) | 141 (15%) |  |
| Not assessed |  | 257 (14%) | 132 (14%) | 125 (13%) |  |
| Missing |  | 12 | 6 | 6 |  |
| ^1^n (%); Mean (SD), For disability, Z-scores corresponds to: ODI, mean = 35.3, SD = 15.5 & NDI, mean = 35.6, SD = 15.3 | | | | |  |
|  | | | | | |

#### Table S1.2: SMS summary

| Measure | Feedback | No feedback |
| --- | --- | --- |
| Replies | 55.6 ± 8.2 (range 24–60) | 55.5 ± 8.4 (range 24–60) |
| Missing | 4.4 ± 8.2 (range 0–36) | 4.5 ± 8.4 (range 0–36) |
| Proportion replied (%) | 92.7 ± 13.6 (range 40–100) | 92.5 ± 14.0 (range 40–100) |
| Proportion missing (%) | 7.3 ± 13.6 (range 0–60) | 7.5 ± 14.0 (range 0–60) |

## Supplementary file 2

#### Table S2.1: Retention analysis

| **Baseline characteristic** | **Total population**  N = 2448 | **100% response rate**  N = 902 | **60% response rate** N = 1768 | **40% response rate** N = 1883 | **<40% response rate (excluded)** N = 565 |
| --- | --- | --- | --- | --- | --- |
| **Pain region** |  |  |  |  |  |
| Low back pain | 2,081 (85%) | 779 (87%) | 1,518 (86%) | 1,616 (86%) | 465 (83%) |
| Neck/mid-back pain | 354 (15%) | 119 (13%) | 241 (14%) | 257 (14%) | 97 (17%) |
| Missing | 13 | 4 | 9 | 10 | 3 |
| **Sex** |  |  |  |  |  |
| Female | 1,321 (54%) | 488 (54%) | 953 (54%) | 1,019 (54%) | 302 (53%) |
| Male | 1,127 (46%) | 414 (46%) | 815 (46%) | 864 (46%) | 263 (47%) |
| **Age (years)** | 59 (14) | 62 (12) | 61 (13) | 60 (13) | 55 (16) |
| **Body mass index (kg/m²)** | 28.1 (5.5) | 28.5 (5.8) | 28.2 (5.5) | 28.2 (5.6) | 27.7 (5.1) |
| Missing | 36 | 6 | 18 | 20 | 16 |
| **Education level** |  |  |  |  |  |
| Basic | 722 (32%) | 273 (32%) | 507 (31%) | 553 (32%) | 169 (33%) |
| Middle | 1,220 (54%) | 446 (53%) | 903 (55%) | 954 (55%) | 266 (52%) |
| Higher | 321 (14%) | 125 (15%) | 232 (14%) | 243 (14%) | 78 (15%) |
| Missing | 185 | 58 | 126 | 133 | 52 |
| **Sick leave past 3 months** |  |  |  |  |  |
| Yes | 616 (27%) | 191 (23%) | 416 (26%) | 447 (26%) | 169 (33%) |
| No | 748 (33%) | 256 (31%) | 533 (33%) | 572 (33%) | 176 (34%) |
| Not assessed | 887 (39%) | 374 (46%) | 680 (42%) | 715 (41%) | 172 (33%) |
| Missing | 197 | 81 | 139 | 149 | 48 |
| **Regional pain (0–10)** | 6 (2) | 6 (2) | 6 (2) | 6 (2) | 6 (2) |
| Missing | 14 | 4 | 9 | 10 | 4 |
| **Extremity pain (0–10)** | 5 (3) | 5 (3) | 5 (3) | 5 (3) | 5 (3) |
| Missing | 14 | 4 | 9 | 10 | 4 |
| **Disability (Z-score)** | 0.00 (1.00) | -0.02 (0.99) | -0.01 (0.98) | -0.01 (0.98) | 0.03 (1.06) |
| Missing | 15 | 4 | 10 | 11 | 4 |
| **STarT Back risk group** |  |  |  |  |  |
| High risk | 1,083 (45%) | 363 (41%) | 758 (43%) | 815 (44%) | 268 (48%) |
| Medium risk | 629 (26%) | 256 (29%) | 481 (27%) | 509 (27%) | 120 (21%) |
| Low risk | 367 (15%) | 158 (18%) | 277 (16%) | 290 (15%) | 77 (14%) |
| Not assessed | 354 (15%) | 119 (13%) | 241 (14%) | 257 (14%) | 97 (17%) |
| Missing | 15 | 6 | 11 | 12 | 3 |
| ^1^n (%); Mean (SD) | | | | | |

## Supplementary file 3

#### Figure S3.1: Model check Root Mean Square of Successive Differences

#### Figure S3.2: Model check Standard deviation

#### Figure S3.3: Model check Mean Absolute Successive Differences

#### Figure S3.4: Model check Root Mean Square Error of Residuals

#### Figure S3.5: Model check Proportion of Weeks with ≥1-Point Change

#### Figure S3.6: Model check Proportion of Weeks with ≥2-Point Change

*Dispersion ratio for a-priori SMS retention analysis*

15.46705

## Supplementary file 4

#### Table S4.1: Group differences for RSME with higher orders time trends

| **Trend specification** | **Mean difference (95% CI)** | **P-value** |
| --- | --- | --- |
| Linear (primary) | -0.014 (-0.059, 0.032) | 0.555 |
| Quadratic (secondary) | -0.017 (-0.059, 0.025) | 0.424 |
| Cubic (secondary) | -0.020 (-0.060, 0.020) | 0.320 |

#### Table S4.2: Regression results – sensitivity for inclusion

| **Outcome** | **Model** | **Primary (40%)** | **Secondary (60%)** | **Secondary (Complete Case)** |
| --- | --- | --- | --- | --- |
| **Root Mean Square of Successive Differences** | **Adjusted** | **-0.054 (-0.103, -0.004) (p = 0.034)** | **-0.063 (-0.113, -0.013) (p = 0.014)** | **-0.089 (-0.160, -0.019) (p = 0.013)** |
|  | **Unadjusted** | **-0.039 (-0.086, 0.008) (p = 0.103)** | **-0.044 (-0.091, 0.004) (p = 0.070)** | **-0.052 (-0.119, 0.014) (p = 0.121)** |
| Standard Deviation of Weekly Pain Intensity | Adjusted | -0.030 (-0.090, 0.031) (p = 0.336) | -0.026 (-0.088, 0.037) (p = 0.418) | -0.042 (-0.129, 0.045) (p = 0.344) |
|  | Unadjusted | -0.018 (-0.075, 0.040) (p = 0.546) | -0.011 (-0.070, 0.048) (p = 0.711) | -0.027 (-0.108, 0.054) (p = 0.514) |
| Mean Absolute Successive Differences | Adjusted | -0.044 (-0.084, -0.005) (p = 0.028) | -0.053 (-0.093, -0.012) (p = 0.011) | -0.068 (-0.125, -0.012) (p = 0.018) |
|  | Unadjusted | -0.034 (-0.072, 0.004) (p = 0.078) | -0.039 (-0.077, -0.000) (p = 0.048) | -0.043 (-0.097, 0.011) (p = 0.115) |
| Root Mean Square Error of Residuals | Adjusted | -0.023 (-0.072, 0.026) (p = 0.353) | -0.024 (-0.074, 0.026) (p = 0.346) | -0.046 (-0.115, 0.023) (p = 0.188) |
|  | Unadjusted | -0.014 (-0.059, 0.032) (p = 0.555) | -0.012 (-0.059, 0.035) (p = 0.613) | -0.026 (-0.091, 0.038) (p = 0.422) |
| Proportion of Weeks with ≥1-Point Change | Adjusted | -0.011 (-0.035, 0.013) (p = 0.386) | -0.013 (-0.038, 0.012) (p = 0.320) | -0.018 (-0.053, 0.017) (p = 0.311) |
|  | Unadjusted | -0.009 (-0.032, 0.013) (p = 0.418) | -0.010 (-0.033, 0.013) (p = 0.400) | -0.015 (-0.047, 0.018) (p = 0.379) |
| Proportion of Weeks with ≥2-Point Change | Adjusted | -0.011 (-0.028, 0.006) (p = 0.207) | -0.009 (-0.027, 0.008) (p = 0.290) | -0.017 (-0.041, 0.007) (p = 0.171) |
|  | Unadjusted | -0.007 (-0.023, 0.009) (p = 0.401) | -0.005 (-0.021, 0.012) (p = 0.591) | -0.013 (-0.035, 0.009) (p = 0.258) |
| Weeks with missing data | Adjusted | 0.950 (0.776, 1.162) (p = 0.612) | 0.978 (0.801, 1.195) (p = 0.829) |  |
|  | Unadjusted | 0.972 (0.806, 1.172) (p = 0.764) | 0.969 (0.806, 1.166) (p = 0.740) |  |

# R script used for analysis

R script used for analysis

#libraries ####

library(tidyverse)

library(gtsummary)

library(scales)

library(gt)

library(broom)

library(patchwork)

library(ggbeeswarm)	# for nicer jitter (quasirandom)

library(scales)

library(flextable)

library(officer)

library(lcmm)

library(readxl)

library(glue)

library(MASS)

library(conflicted)

conflict_prefer("select", "dplyr")

conflict_prefer("filter", "dplyr")

library(lmtest)

library(easystats)

library(colorspace)

library(ggpubr)

conflicts_prefer(flextable::font)

library(ggsankey)

library(nnet)

library(mice)

scipen=999

\# Load datasets ####

df.sms_system <- readRDS("01_rawdata/sms_data_completed.RDS") %>%

select(record_id=id,week,spine_area,job_status, sms_date, status) %>%

arrange(record_id,week)

ID <- readRDS("01_rawdata/READY/ID.RDS")

RCT_GROUPS<-read.csv("01_rawdata/READY/Pathback_RCT_blinding.csv",sep=";") %>%

mutate(record_id=as.character(record_id),

group=as.factor(lige_ulige),.keep="unused") %>%

mutate(group_unblind=fct_recode(group,

"No feedback"="A",

"Feedback"="B"))

MIRD <- readRDS("01_rawdata/READY/MIRD.RDS")

SMS_PAIN_SL <- readRDS("01_rawdata/READY/SMS_PAIN_SL.RD")

\## Calculate include rates

INCLUDE <- SMS_PAIN_SL %>%

group_by(record_id) %>%

summarise(

n_weeks = n_distinct(week),

n_valid = sum(!is.na(painlevel)),

prop_valid = n_valid / n_weeks, # proportion of valid replies

include100 = n_valid == n_weeks, # all 60 answered

include60 = n_valid >= 36, # ≥ 60% answered

include40 = n_valid >= 24, # ≥ 40% answered

.groups = "drop"

)

\## SMS data for RCT ####

df.sms.rct<-

SMS_PAIN_SL %>%

left_join(INCLUDE, by = "record_id") %>%

filter(include40) %>%

mutate(week = readr::parse_number(week)) %>%

select(record_id,week,painlevel) %>%

left_join(RCT_GROUPS)

#Inclusion period 05-02-2023 to 19-05-2024

\# Flowchart numbers ####

SMS_PAIN_SL %>%

\# merge randomized group info

left_join(RCT_GROUPS, by = "record_id") %>%

\# extract numeric week number

mutate(week_num = readr::parse_number(week)) %>%

\# summarise participant-level response pattern

group_by(record_id, group) %>%

summarise(

n_weeks = n_distinct(week_num),

n_valid = sum(!is.na(painlevel)),

.groups = "drop"

) %>%

\# classify eligibility thresholds (40% = 24 weeks, 60% = 36 weeks)

mutate(

eligible_40 = n_valid >= 24,

eligible_60 = n_valid >= 36

) %>%

\# summarise counts per group and overall

bind_rows(

summarise(., group = "Overall", across(c(n_valid, eligible_40, eligible_60), ~ .x))

) %>%

group_by(group) %>%

summarise(

n_total = n(),

n_any = sum(n_valid > 0),

n_40 = sum(eligible_40),

n_60 = sum(eligible_60),

p_any = n_any / n_total * 100,

p_40 = n_40 / n_total * 100,

p_60 = n_60 / n_total * 100,

.groups = "drop"

)

df.sms_system %>%

filter(str_detect(status, regex("drop.?out", ignore_case = TRUE))) %>%

distinct(record_id,status) %>%

mutate(record_id=as.character(record_id)) %>%

left_join(RCT_GROUPS) %>%

group_by(group) %>%

mutate(drops=n()) %>%

group_by(status,group) %>%

count() %>%

arrange(group)

#check

INCLUDE %>%

left_join(RCT_GROUPS) %>%

group_by(group) %>%

summarise(

n_total = n(),

n_100 = sum(include100),

n_60 = sum(include60),

n_40 = sum(include40)

) %>% gt()

\# Demographic table ####

MIRD %>%

left_join(RCT_GROUPS, by = "record_id") %>%

left_join(INCLUDE) %>%

filter(include40) %>%

select(

group, region, sex, age, BMI, education,

sl3mon, paintyp, paintyp_extremity,

disability, SBT

) %>%

tbl_summary(

by = group,

missing = "ifany",

missing_text = "Missing",

statistic = list(

all_continuous() ~ "{mean} ({sd})",

all_categorical() ~ "{n} ({p}%)"

),

label = list(

region ~ "Pain region",

sex ~ "Sex",

age ~ "Age (years)",

BMI ~ "Body mass index (kg/m²)",

education ~ "Education level",

sl3mon ~ "Sick leave past 3 months",

paintyp ~ "Regional pain (0–10)",

paintyp_extremity ~ "Extremity pain (0–10)",

disability ~ "Disability (Z-score)",

SBT ~ "STarT Back risk group"

)

) %>%

add_overall() %>%

add_n() %>%

bold_labels() %>%

as_flex_table() %>%

save_as_docx(

path = "A_Papers/Nested_RCT/PAPER1/B_FIGURES_TABLES/baseline_table.docx"

)

\## What are the SDs for disability

MIRD %>%

left_join(RCT_GROUPS, by = "record_id") %>%

left_join(INCLUDE) %>%

filter(include40) %>%

pivot_longer(cols = c(ODI, NDI), names_to = "scale", values_to = "score") %>%

group_by(region, scale) %>%

summarise(mean = mean(score, na.rm = TRUE),

sd = sd(score, na.rm = TRUE),

.groups = "drop") %>%

na.omit() %>%

gt()

\# SMS retention ####

SMS_PAIN_SL %>%

left_join(INCLUDE, by = "record_id") %>%

left_join(RCT_GROUPS) %>%

filter(include40) %>%

group_by(record_id, group) %>%

summarise(

n_weeks = n_distinct(week),

n_replies = sum(!is.na(painlevel)),

n_missing = sum(is.na(painlevel)),

prop_replied = n_replies / n_weeks * 100,

prop_missing = n_missing / n_weeks * 100,

.groups = "drop"

) %>%

group_by(group) %>%

summarise(

Replies = sprintf("%.1f ± %.1f (range %d–%d)", mean(n_replies), sd(n_replies), min(n_replies), max(n_replies)),

Missing = sprintf("%.1f ± %.1f (range %d–%d)", mean(n_missing), sd(n_missing), min(n_missing), max(n_missing)),

`Proportion replied (%)` = sprintf("%.1f ± %.1f (range %.0f–%.0f)", mean(prop_replied), sd(prop_replied), min(prop_replied), max(prop_replied)),

`Proportion missing (%)` = sprintf("%.1f ± %.1f (range %.0f–%.0f)", mean(prop_missing), sd(prop_missing), min(prop_missing), max(prop_missing))

) %>%

pivot_longer(-group, names_to = "Measure", values_to = "Value") %>%

pivot_wider(names_from = group, values_from = Value) %>%

flextable() %>%

set_header_labels(Measure = "Measure", A = "Group A", B = "Group B") %>%

autofit() %>%

bold(part = "header") %>%

fontsize(size = 10, part = "all") %>%

theme_booktabs() %>%

align(align = "center", part = "all")%>%

save_as_docx(path = "A_Papers/Nested_RCT/PAPER1/B_FIGURES_TABLES/sms_summary.docx")

\# Dropout analysis ####

\# Identify "None" participants first

none_ids <- INCLUDE %>%

select(record_id, contains("include")) %>%

mutate(across(contains("include"), ~replace_na(., FALSE))) %>%

filter(!include100 & !include60 & !include40) %>%

pull(record_id)

\# Create long dataset (participants may appear in several inclusion groups)

df_long <- MIRD %>%

left_join(

INCLUDE %>%

select(record_id, contains("include")) %>%

pivot_longer(cols = -record_id)

) %>%

mutate(

inclusion_group = case_when(

name == "include100" & value ~ "Include 100",

name == "include60" & value ~ "Include 60",

name == "include40" & value ~ "Include 40",

TRUE ~ NA_character_

)

)

\# Add "None" group

df_summary <- bind_rows(

df_long %>% filter(!is.na(inclusion_group)),

MIRD %>%

filter(record_id %in% none_ids) %>%

mutate(inclusion_group = "None")

)

\# Factor levels for order

df_summary <- df_summary %>%

mutate(

inclusion_group = factor(

inclusion_group,

levels = c("Include 100", "Include 60", "Include 40", "None")

)

)

\# Create dataset for unique participants (true "Overall" group)

df_overall <- MIRD %>%

mutate(inclusion_group = "Overall")

\# Combine both

df_final <- bind_rows(df_overall, df_summary) %>%

mutate(inclusion_group=fct_relevel(inclusion_group,

"Overall","Include 100","Include 60", "Include 40","None"))

\# Compute group sizes manually (unique participants)

group_n <- df_final %>%

distinct(record_id, inclusion_group) %>%

count(inclusion_group) %>%

mutate(label = glue("N = {formatC(n, format = 'd', big.mark = ',')}"))

\# Build the table

df_final %>%

mutate(

inclusion_group = fct_relevel(inclusion_group,

"Overall", "Include 100", "Include 60", "Include 40", "None")

) %>%

select(

inclusion_group,

region, sex, age, BMI, education,

sl3mon, paintyp, paintyp_extremity,

disability, SBT

) %>%

tbl_summary(

by = inclusion_group,

missing = "ifany",

missing_text = "Missing",

statistic = list(

all_continuous() ~ "{mean} ({sd})",

all_categorical() ~ "{n} ({p}%)"

),

label = list(

region ~ "Pain region",

sex ~ "Sex",

age ~ "Age (years)",

BMI ~ "Body mass index (kg/m²)",

education ~ "Education level",

sl3mon ~ "Sick leave past 3 months",

paintyp ~ "Regional pain (0–10)",

paintyp_extremity ~ "Extremity pain (0–10)",

disability ~ "Disability (Z-score)",

SBT ~ "STarT Back risk group"	)

) %>%

bold_labels() %>%

modify_header(

label ~ "**Baseline characteristic**",

stat_1 ~ glue("**Total population**<br><sub>N = {group_n$n[group_n$inclusion_group == 'Overall']}</sub>"),

stat_2 ~ glue("**100% response rate**<br><sub>N = {group_n$n[group_n$inclusion_group == 'Include 100']}</sub>"),

stat_3 ~ glue("**60% response rate**<br><sub>N = {group_n$n[group_n$inclusion_group == 'Include 60']}</sub>"),

stat_4 ~ glue("**40% response rate**<br><sub>N = {group_n$n[group_n$inclusion_group == 'Include 40']}</sub>"),

stat_5 ~ glue("**<40% response rate (excluded)**<br><sub>N = {group_n$n[group_n$inclusion_group == 'None']}</sub>")

) %>%

as_flex_table() %>%

save_as_docx(

path = "A_Papers/Nested_RCT/PAPER1/B_FIGURES_TABLES/dropout_table.docx"

)

\# Calculations ####

person_metrics <- df.sms.rct %>%

mutate(week = as.integer(week)) %>%	# ensure numeric week variable

arrange(record_id, week) %>%

group_by(record_id, group,group_unblind) %>%

group_modify(~{

d <- .x

\# 1. Filter valid pain values

x <- d$painlevel[!is.na(d$painlevel)]

\# 2. Skip if fewer than 2 observations

if (length(x) < 2) {

return(tibble(

n_weeks = sum(!is.na(d$painlevel)),

mean_pain = mean(d$painlevel, na.rm = TRUE),

sd_pain = sd(d$painlevel, na.rm = TRUE),

rmssd = NA_real_,

masd = NA_real_,

rmse_trend = NA_real_,

pac1 = NA_real_,

pac2 = NA_real_

))

}

\# 3. Successive differences

diff_vec <- diff(x)

\# 4. RMSE around linear trend (requires ≥3 time points)

d_fit <- d %>% filter(!is.na(painlevel), !is.na(week))

rmse_trend <- if (nrow(d_fit) >= 3) {

fit <- lm(painlevel ~ week, data = d_fit)

sqrt(mean(residuals(fit)^2))

} else NA_real_

\# 5. Deviation from personal mean

mean_i <- mean(x)

dev <- abs(x - mean_i)

\# 6. Return all metrics

tibble(

n_weeks	= sum(!is.na(d$painlevel)),

mean_pain = mean(x),

sd_pain	= sd(x),

rmssd	= sqrt(mean(diff_vec^2)),

masd	= mean(abs(diff_vec)),

rmse_trend = rmse_trend,

pac1	= mean(dev >= 1),

pac2	= mean(dev >= 2)

)

}) %>%

ungroup() %>%

mutate(group_unblind=fct_relevel(group_unblind,

c("Feedback","No feedback")))

\# Report findings descriptively ####

\## helper for variability metrics (RMSSD, SD, RMSE, MASD)

plot_var <- function(data, metric, ylab) {

ggplot(data, aes(x = group_unblind, y = .data[[metric]], fill = group_unblind)) +

geom_violin(trim = FALSE, alpha = 0.4, color = NA) +

geom_boxplot(width = 0.15, outlier.shape = NA, alpha = 0.7) +

geom_quasirandom(width = 0.25, alpha = 0.35, size = 0.1) +

scale_fill_manual(values = c("#4B9CD3", "#E07B39")) +

coord_cartesian(ylim = c(0, 5)) +

labs(x = NULL, y = ylab) +

theme_minimal(base_size = 14) +

theme(

legend.position = "none",

panel.grid.major.x = element_blank(),

axis.title.x = element_blank()

)

}

\## helper for PAC metrics (PAC1, PAC2)

plot_pac <- function(data, metric, ylab) {

ggplot(data, aes(x = group_unblind, y = .data[[metric]], fill = group_unblind)) +

geom_violin(trim = FALSE, alpha = 0.4, color = NA) +

geom_boxplot(width = 0.15, outlier.shape = NA, alpha = 0.7) +

geom_quasirandom(width = 0.25, alpha = 0.35, size = 0.1) +

scale_fill_manual(values = c("#4B9CD3", "#E07B39")) +

coord_cartesian(ylim = c(0, 1)) +

labs(x = NULL, y = ylab) +

theme_minimal(base_size = 14) +

theme(

legend.position = "none",

panel.grid.major.x = element_blank(),

axis.title.x = element_blank())+

scale_y_continuous(labels=scales::percent)

}

\## create plots

p_rmssd <- plot_var(person_metrics, "rmssd", "RMSSD")

p_sd	<- plot_var(person_metrics, "sd_pain", "SD")

p_rmse <- plot_var(person_metrics, "rmse_trend", "RMSE")

p_masd <- plot_var(person_metrics, "masd", "MASD")

p_pac1 <- plot_pac(person_metrics, "pac1", "PAC1")

p_pac2 <- plot_pac(person_metrics, "pac2", "PAC2")

\## combine

final_plot <- (

p_rmssd /

(p_sd | p_rmse | p_masd) /

(p_pac1 | p_pac2)

) +

plot_annotation(

theme = theme(

plot.title = element_text(size = 18, face = "bold"),

plot.subtitle = element_text(size = 13)

)

)

final_plot

ggsave("A_Papers/Nested_RCT/B_FIGURES_TABLES/descriptive.png",dpi=300,width=20,height=30,units="cm")

\# calculations ####

person_retention <- df.sms.rct %>%

group_by(record_id,group,group_unblind) %>%

summarise(

n_weeks = n_distinct(week),

n_missing = sum(is.na(painlevel)),

n_valid = sum(!is.na(painlevel)),

prop_missing = n_missing / n_weeks,

.groups = "drop"

) %>%

mutate(group_unblind=fct_relevel(group_unblind,

c("Feedback","No feedback")))

\# Mean and SDs ####

person_retention %>%

group_by(group_unblind) %>%

summarise(mean_n=mean(n_missing), # mean weeks with missing data

sd_n=sd(n_missing), # sd weeks with missing data

\# the same just proportions

mean_prop=mean(prop_missing),

sd_prop=sd(prop_missing))

\# Plots ####

ggplot(person_retention, aes(x = group_unblind, y = n_missing, fill = group_unblind)) +

geom_violin(trim = FALSE, alpha = 0.4, color = NA) +

geom_boxplot(width = 0.15, outlier.shape = NA, alpha = 0.7) +

geom_quasirandom(width = 0.25, alpha = 0.35, size = 1) +

scale_fill_manual(values = c("#4B9CD3", "#E07B39")) +

coord_cartesian(ylim = c(0, 36)) +	# since max possible weeks = 60

labs(

x = NULL,

y = "Number of missing weekly responses",

) +

theme_minimal(base_size = 14) +

theme(

legend.position = "none",

panel.grid.major.x = element_blank(),

plot.title = element_text(size = 18, face = "bold"),

plot.subtitle = element_text(size = 13)

)+

scale_y_continuous(breaks = c(0,6,12,18,24,30,36))+

ggplot(person_retention, aes(x = group_unblind, y = prop_missing, fill = group_unblind)) +

geom_violin(trim = FALSE, alpha = 0.4, color = NA) +

geom_boxplot(width = 0.15, outlier.shape = NA, alpha = 0.7) +

geom_quasirandom(width = 0.25, alpha = 0.35, size = 0.8) +

scale_fill_manual(values = c("#4B9CD3", "#E07B39")) +

coord_cartesian(ylim = c(0, 0.6)) +

scale_y_continuous(labels = scales::percent,

breaks=c(0.0,.1,.2,.3,.4,.5,.6))+

labs(

x = NULL,

y = "Proportion of missing weeks",

) +

theme_minimal(base_size = 14) +

theme(legend.position = "none")

ggsave("A_Papers/Nested_RCT/B_FIGURES_TABLES/missingweeks.png",dpi=300,width=20,height=15,units="cm")

\# Create df for analysis ####

df_analysis <- person_metrics %>%

left_join(person_retention %>% select(record_id, n_missing),

by = "record_id") %>%

left_join(MIRD %>%

select(record_id, region, sex, age, BMI, education,

sl3mon, paintyp, paintyp_extremity,

disability, SBT

))

\## define outcomes and covariates ####

outcomes <- c("rmssd", "sd_pain", "rmse_trend", "masd", "pac1","pac2")

outcome_sms<- c("n_missing")

covariates <- c(

"region", "sex", "age", "BMI", "education",

"sl3mon", "paintyp", "paintyp_extremity", "disability", "SBT"

)

\## run all models ####

results_all <- outcomes %>%

map_dfr(function(v) {

\# build formulas

form_unadj <- as.formula(paste(v, "~ group"))

form_adj	<- as.formula(paste(v, "~ group +", paste(covariates, collapse = " + ")))

\# fit models

m1 <- lm(form_unadj, data = df_analysis)

m2 <- lm(form_adj, data = df_analysis)

\# extract results for group effect

bind_rows(

tidy(m1, conf.int = TRUE) %>%

filter(term == "groupB") %>%

mutate(model = "Unadjusted"),

tidy(m2, conf.int = TRUE) %>%

filter(term == "groupB") %>%

mutate(model = "Adjusted")

) %>%

mutate(outcome = v)

}) %>%

select(outcome, model, estimate, conf.low, conf.high, p.value) %>%

mutate(

across(estimate:conf.high, round, 3),

p.value = pvalue(p.value)

)

results_all_sms <- outcome_sms %>%

map_dfr(function(v) {

form_unadj <- as.formula(paste(v, "~ group"))

form_adj	<- as.formula(paste(v, "~ group +", paste(covariates, collapse = " + ")))

\# Fit negative binomial models

m1 <- glm.nb(form_unadj, data = df_analysis)

m2 <- glm.nb(form_adj, data = df_analysis)

\# Extract exponentiated coefficients (rate ratios)

bind_rows(

tidy(m1, conf.int = TRUE, exponentiate = TRUE) %>%

filter(term == "groupB") %>%

mutate(model = "Unadjusted"),

tidy(m2, conf.int = TRUE, exponentiate = TRUE) %>%

filter(term == "groupB") %>%

mutate(model = "Adjusted")

) %>%

mutate(outcome = v)

}) %>%

select(outcome, model, estimate, conf.low, conf.high, p.value) %>%

mutate(across(estimate:conf.high, round, 3),

p.value = pvalue(p.value))

\## tabulated results ####

results_all_TAB_PRE<-

results_all %>%

mutate(

outcome = recode(outcome,

rmssd = "Root Mean Square of Successive Differences",

sd_pain = "Standard Deviation of Weekly Pain Intensity",

rmse_trend = "Root Mean Square Error of Residuals",

masd = "Mean Absolute Successive Differences",

pac1 = "Proportion of Weeks with ≥1-Point Change",

pac2 = "Proportion of Weeks with ≥2-Point Change",

),

estimate_CI = sprintf("%.3f (%.3f, %.3f)", estimate, conf.low, conf.high)

) %>%

select(

Outcome = outcome,

Model = model,

`Mean difference (95% CI)` = estimate_CI,

`p-value` = p.value

) %>%

mutate(Outcome = factor(Outcome, levels = c(

"Root Mean Square of Successive Differences",

"Standard Deviation of Weekly Pain Intensity",

"Mean Absolute Successive Differences",

"Root Mean Square Error of Residuals",

"Proportion of Weeks with ≥1-Point Change",

"Proportion of Weeks with ≥2-Point Change"

))) %>%

mutate(Model = fct_relevel(Model,"Unadjusted","Adjusted")) %>%

arrange(Outcome, Model)

results_sms_TAB_PRE<-

results_all_sms %>%

mutate(

outcome = recode(outcome_sms,

n_missing = "Weeks with missing data"),

estimate_CI = sprintf("%.3f (%.3f, %.3f)", estimate, conf.low, conf.high)

) %>%

select(

Outcome = outcome,

Model = model,

`Mean difference (95% CI)` = estimate_CI,

`p-value` = p.value

) %>%

mutate(Model = fct_relevel(Model,"Unadjusted","Adjusted")) %>%

arrange(Outcome, Model)

\# Create the table ####

RESULTS_TABLE<-

results_all_TAB_PRE %>%

rbind(results_sms_TAB_PRE) %>%

flextable() %>%

merge_v(j = 1) %>%	# collapse repeated outcome labels

align(align = "center", part = "all") %>%

autofit() %>%

fontsize(part = "all", size = 11) %>%

font(part = "all", fontname = "Calibri") %>%

border_remove() %>%

border_outer(part = "all", border = officer::fp_border(color = "black", width = 1)) %>%

border_inner_h(part = "body", border = officer::fp_border(color = "gray", width = 0.5)) %>%

border_inner_v(part = "body", border = officer::fp_border(color = "gray", width = 0.5)) %>%

\# Highlight RMSSD row block

bg(i = ~ Outcome == "Root Mean Square of Successive Differences", bg = "#f2f2f2") %>%

bold(i = ~ Outcome == "Root Mean Square of Successive Differences", bold = TRUE) %>%

bg(i = ~ Outcome == "Root Mean Square of Successive Differences", bg = "#f2f2f2") %>%

bold(i = ~ Outcome == "Root Mean Square of Successive Differences", bold = TRUE)

\# Save to Word

save_as_docx(RESULTS_TABLE,path = "A_Papers/Nested_RCT/B_FIGURES_TABLES/regression_results.docx")

\# model checks ####

\## over disperson for posisson ####

sms_model_check <- glm(n_missing ~ group, data = df_analysis, family = quasipoisson)

dispersion_ratio <- sum(residuals(sms_model_check, type = "pearson")^2) / sms_model_check$df.residual

dispersion_ratio

\# needed to redo this

\## Homoscedasticity (Breusch-Pagan test) ####

outcomes <- c("rmssd", "sd_pain", "rmse_trend", "masd", "pac1", "pac2")

map_dfr(outcomes, function(v) {

form <- as.formula(paste(v, "~ group"))

m <- lm(form, data = df_analysis)

tibble(

outcome = v,

bp_p = bptest(m)$p.value

)

})

\## overall diagnostics ####

lm(rmssd ~ group, data = df_analysis) %>% check_model()

lm(sd_pain ~ group, data = df_analysis) %>% check_model()

lm(rmse_trend ~ group, data = df_analysis) %>% check_model()

lm(masd ~ group, data = df_analysis) %>% check_model()

lm(pac1 ~ group, data = df_analysis) %>% check_model()

lm(pac2 ~ group, data = df_analysis) %>% check_model()

\# RMSE ####

\# Define helper for computing RMSE given model formula

compute_rmse <- function(formula, data) {

model <- lm(formula, data = data)

sqrt(mean(residuals(model)^2, na.rm = TRUE))

}

\# Compute RMSEs per person and trend specification

rmse_trends <- df.sms.rct %>%

group_by(record_id, group) %>%

summarise(

rmse_linear = compute_rmse(painlevel ~ week, cur_data()),

rmse_quadratic = compute_rmse(painlevel ~ week + I(week^2), cur_data()),

rmse_cubic = compute_rmse(painlevel ~ week + I(week^2) + I(week^3), cur_data()),

.groups = "drop"

) %>%

pivot_longer(starts_with("rmse_"), names_to = "trend", values_to = "RMSE")

\# Compare group differences for each trend

rmse_results <- rmse_trends %>%

group_split(trend) %>%

map_dfr(~ {

m <- lm(RMSE ~ group, data = .x)

tidy(m, conf.int = TRUE) %>%

filter(term == "groupB") %>%

mutate(trend = unique(.x$trend))

}) %>%

mutate(

trend = recode(trend,

rmse_linear = "Linear (primary)",

rmse_quadratic = "Quadratic (secondary)",

rmse_cubic = "Cubic (secondary)"),

estimate_CI = sprintf("%.3f (%.3f, %.3f)", estimate, conf.low, conf.high),

across(estimate:conf.high, round, 3),

p.value = scales::pvalue(p.value)

) %>%

mutate(trend=fct_relevel(trend,

"Linear (primary)","Quadratic (secondary)","Cubic (secondary)")) %>%

arrange(trend) %>%

select(`Trend specification` = trend, `Mean difference (95% CI)` = estimate_CI, `p-value` = p.value)

\# Pretty table for Word

flextable(rmse_results) %>%

align(align = "center", part = "all") %>%

autofit() %>%

font(fontname = "Calibri", part = "all") %>%

fontsize(size = 11, part = "all") %>%

border_outer(border = fp_border(color = "black", width = 1)) %>%

border_inner_h(border = fp_border(color = "gray", width = 0.5)) %>%

border_inner_v(border = fp_border(color = "gray", width = 0.5)) %>%

save_as_docx(path = "A_Papers/Nested_RCT/B_FIGURES_TABLES/RMSE_trend_sensitivity.docx")

\# 60% Retention ####

\## create data ####

df.sms.rct60<-

SMS_PAIN_SL %>%

left_join(INCLUDE, by = "record_id") %>%

filter(include60) %>%

mutate(week = readr::parse_number(week)) %>%

select(record_id,week,painlevel) %>%

left_join(RCT_GROUPS)

\## person metrics

person_metrics60 <- df.sms.rct60 %>%

mutate(week = as.integer(week)) %>%	# ensure numeric week variable

arrange(record_id, week) %>%

group_by(record_id, group) %>%

group_modify(~{

d <- .x

\# 1. Filter valid pain values

x <- d$painlevel[!is.na(d$painlevel)]

\# 2. Skip if fewer than 2 observations

if (length(x) < 2) {

return(tibble(

n_weeks = sum(!is.na(d$painlevel)),

mean_pain = mean(d$painlevel, na.rm = TRUE),

sd_pain = sd(d$painlevel, na.rm = TRUE),

rmssd = NA_real_,

masd = NA_real_,

rmse_trend = NA_real_,

pac1 = NA_real_,

pac2 = NA_real_

))

}

\# 3. Successive differences

diff_vec <- diff(x)

\# 4. RMSE around linear trend (requires ≥3 time points)

d_fit <- d %>% filter(!is.na(painlevel), !is.na(week))

rmse_trend <- if (nrow(d_fit) >= 3) {

fit <- lm(painlevel ~ week, data = d_fit)

sqrt(mean(residuals(fit)^2))

} else NA_real_

\# 5. Deviation from personal mean

mean_i <- mean(x)

dev <- abs(x - mean_i)

\# 6. Return all metrics

tibble(

n_weeks	= sum(!is.na(d$painlevel)),

mean_pain = mean(x),

sd_pain	= sd(x),

rmssd	= sqrt(mean(diff_vec^2)),

masd	= mean(abs(diff_vec)),

rmse_trend = rmse_trend,

pac1	= mean(dev >= 1),

pac2	= mean(dev >= 2)

)

}) %>%

ungroup()

\# regression

df_analysis60 <- person_metrics60 %>%

left_join(person_retention %>% select(record_id, n_missing),

by = "record_id") %>%

left_join(MIRD %>%

select(record_id, region, sex, age, BMI, education,

sl3mon, paintyp, paintyp_extremity,

disability, SBT

))

\## run all models ####

results_all60 <- outcomes %>%

map_dfr(function(v) {

\# build formulas

form_unadj <- as.formula(paste(v, "~ group"))

form_adj	<- as.formula(paste(v, "~ group +", paste(covariates, collapse = " + ")))

\# fit models

m1 <- lm(form_unadj, data = df_analysis60)

m2 <- lm(form_adj, data = df_analysis60)

\# extract results for group effect

bind_rows(

tidy(m1, conf.int = TRUE) %>%

filter(term == "groupB") %>%

mutate(model = "Unadjusted"),

tidy(m2, conf.int = TRUE) %>%

filter(term == "groupB") %>%

mutate(model = "Adjusted")

) %>%

mutate(outcome = v)

}) %>%

select(outcome, model, estimate, conf.low, conf.high, p.value) %>%

mutate(

across(estimate:conf.high, round, 3),

p.value = pvalue(p.value)

)

results_all_sms60 <- outcome_sms %>%

map_dfr(function(v) {

form_unadj <- as.formula(paste(v, "~ group"))

form_adj	<- as.formula(paste(v, "~ group +", paste(covariates, collapse = " + ")))

\# Fit negative binomial models

m1 <- glm.nb(form_unadj, data = df_analysis60)

m2 <- glm.nb(form_adj, data = df_analysis60)

\# Extract exponentiated coefficients (rate ratios)

bind_rows(

tidy(m1, conf.int = TRUE, exponentiate = TRUE) %>%

filter(term == "groupB") %>%

mutate(model = "Unadjusted"),

tidy(m2, conf.int = TRUE, exponentiate = TRUE) %>%

filter(term == "groupB") %>%

mutate(model = "Adjusted")

) %>%

mutate(outcome = v)

}) %>%

select(outcome, model, estimate, conf.low, conf.high, p.value) %>%

mutate(across(estimate:conf.high, round, 3),

p.value = pvalue(p.value))

\## tabulated results ####

results_all_TAB_PRE60<-

results_all60 %>%

mutate(

outcome = recode(outcome,

rmssd = "Root Mean Square of Successive Differences",

sd_pain = "Standard Deviation of Weekly Pain Intensity",

rmse_trend = "Root Mean Square Error of Residuals",

masd = "Mean Absolute Successive Differences",

pac1 = "Proportion of Weeks with ≥1-Point Change",

pac2 = "Proportion of Weeks with ≥2-Point Change",

),

estimate_CI = sprintf("%.3f (%.3f, %.3f)", estimate, conf.low, conf.high)

) %>%

select(

Outcome = outcome,

Model = model,

`Mean difference (95% CI)` = estimate_CI,

`p-value` = p.value

) %>%

mutate(Outcome = factor(Outcome, levels = c(

"Root Mean Square of Successive Differences",

"Standard Deviation of Weekly Pain Intensity",

"Mean Absolute Successive Differences",

"Root Mean Square Error of Residuals",

"Proportion of Weeks with ≥1-Point Change",

"Proportion of Weeks with ≥2-Point Change"

))) %>%

mutate(Model = fct_relevel(Model,"Unadjusted","Adjusted")) %>%

arrange(Outcome, Model)

results_sms_TAB_PRE60<-

results_all_sms60 %>%

mutate(

outcome = recode(outcome_sms,

n_missing = "Weeks with missing data"),

estimate_CI = sprintf("%.3f (%.3f, %.3f)", estimate, conf.low, conf.high)

) %>%

select(

Outcome = outcome,

Model = model,

`Mean difference (95% CI)` = estimate_CI,

`p-value` = p.value

) %>%

mutate(Model = fct_relevel(Model,"Unadjusted","Adjusted")) %>%

arrange(Outcome, Model)

\# Create the table ####

RESULTS_TABLE60<-

results_all_TAB_PRE60 %>%

rbind(results_sms_TAB_PRE60) %>%

flextable() %>%

merge_v(j = 1) %>%	# collapse repeated outcome labels

align(align = "center", part = "all") %>%

autofit() %>%

fontsize(part = "all", size = 11) %>%

font(part = "all", fontname = "Calibri") %>%

border_remove() %>%

border_outer(part = "all", border = officer::fp_border(color = "black", width = 1)) %>%

border_inner_h(part = "body", border = officer::fp_border(color = "gray", width = 0.5)) %>%

border_inner_v(part = "body", border = officer::fp_border(color = "gray", width = 0.5)) %>%

\# Highlight RMSSD row block

bg(i = ~ Outcome == "Root Mean Square of Successive Differences", bg = "#f2f2f2") %>%

bold(i = ~ Outcome == "Root Mean Square of Successive Differences", bold = TRUE) %>%

bg(i = ~ Outcome == "Root Mean Square of Successive Differences", bg = "#f2f2f2") %>%

bold(i = ~ Outcome == "Root Mean Square of Successive Differences", bold = TRUE)

\# 100% Complete case ####

\## create data ####

df.sms.rct100<-

SMS_PAIN_SL %>%

left_join(INCLUDE, by = "record_id") %>%

filter(include100) %>%

mutate(week = readr::parse_number(week)) %>%

select(record_id,week,painlevel) %>%

left_join(RCT_GROUPS)

\## person metrics

person_metrics100 <- df.sms.rct100 %>%

mutate(week = as.integer(week)) %>%	# ensure numeric week variable

arrange(record_id, week) %>%

group_by(record_id, group) %>%

group_modify(~{

d <- .x

\# 1. Filter valid pain values

x <- d$painlevel[!is.na(d$painlevel)]

\# 2. Skip if fewer than 2 observations

if (length(x) < 2) {

return(tibble(

n_weeks = sum(!is.na(d$painlevel)),

mean_pain = mean(d$painlevel, na.rm = TRUE),

sd_pain = sd(d$painlevel, na.rm = TRUE),

rmssd = NA_real_,

masd = NA_real_,

rmse_trend = NA_real_,

pac1 = NA_real_,

pac2 = NA_real_

))

}

\# 3. Successive differences

diff_vec <- diff(x)

\# 4. RMSE around linear trend (requires ≥3 time points)

d_fit <- d %>% filter(!is.na(painlevel), !is.na(week))

rmse_trend <- if (nrow(d_fit) >= 3) {

fit <- lm(painlevel ~ week, data = d_fit)

sqrt(mean(residuals(fit)^2))

} else NA_real_

\# 5. Deviation from personal mean

mean_i <- mean(x)

dev <- abs(x - mean_i)

\# 6. Return all metrics

tibble(

n_weeks	= sum(!is.na(d$painlevel)),

mean_pain = mean(x),

sd_pain	= sd(x),

rmssd	= sqrt(mean(diff_vec^2)),

masd	= mean(abs(diff_vec)),

rmse_trend = rmse_trend,

pac1	= mean(dev >= 1),

pac2	= mean(dev >= 2)

)

}) %>%

ungroup()

\# regression

df_analysis100 <- person_metrics100 %>%

left_join(person_retention %>% select(record_id, n_missing),

by = "record_id") %>%

left_join(MIRD %>%

select(record_id, region, sex, age, BMI, education,

sl3mon, paintyp, paintyp_extremity,

disability, SBT

))

\## run all models ####

results_all100 <- outcomes %>%

map_dfr(function(v) {

\# build formulas

form_unadj <- as.formula(paste(v, "~ group"))

form_adj	<- as.formula(paste(v, "~ group +", paste(covariates, collapse = " + ")))

\# fit models

m1 <- lm(form_unadj, data = df_analysis100)

m2 <- lm(form_adj, data = df_analysis100)

\# extract results for group effect

bind_rows(

tidy(m1, conf.int = TRUE) %>%

filter(term == "groupB") %>%

mutate(model = "Unadjusted"),

tidy(m2, conf.int = TRUE) %>%

filter(term == "groupB") %>%

mutate(model = "Adjusted")

) %>%

mutate(outcome = v)

}) %>%

select(outcome, model, estimate, conf.low, conf.high, p.value) %>%

mutate(

across(estimate:conf.high, round, 3),

p.value = pvalue(p.value)

)

\## tabulated results ####

RESULTS_TABLE100<-

results_all100 %>%

mutate(

outcome = recode(outcome,

rmssd = "Root Mean Square of Successive Differences",

sd_pain = "Standard Deviation of Weekly Pain Intensity",

rmse_trend = "Root Mean Square Error of Residuals",

masd = "Mean Absolute Successive Differences",

pac1 = "Proportion of Weeks with ≥1-Point Change",

pac2 = "Proportion of Weeks with ≥2-Point Change",

),

estimate_CI = sprintf("%.3f (%.3f, %.3f)", estimate, conf.low, conf.high)

) %>%

select(

Outcome = outcome,

Model = model,

`Mean difference (95% CI)` = estimate_CI,

`p-value` = p.value

) %>%

mutate(Outcome = factor(Outcome, levels = c(

"Root Mean Square of Successive Differences",

"Standard Deviation of Weekly Pain Intensity",

"Mean Absolute Successive Differences",

"Root Mean Square Error of Residuals",

"Proportion of Weeks with ≥1-Point Change",

"Proportion of Weeks with ≥2-Point Change"

))) %>%

mutate(Model = fct_relevel(Model,"Unadjusted","Adjusted")) %>%

arrange(Outcome, Model) %>%

flextable() %>%

merge_v(j = 1) %>%	# collapse repeated outcome labels

align(align = "center", part = "all") %>%

autofit() %>%

fontsize(part = "all", size = 11) %>%

font(part = "all", fontname = "Calibri") %>%

border_remove() %>%

border_outer(part = "all", border = officer::fp_border(color = "black", width = 1)) %>%

border_inner_h(part = "body", border = officer::fp_border(color = "gray", width = 0.5)) %>%

border_inner_v(part = "body", border = officer::fp_border(color = "gray", width = 0.5)) %>%

\# Highlight RMSSD row block

bg(i = ~ Outcome == "Root Mean Square of Successive Differences", bg = "#f2f2f2") %>%

bold(i = ~ Outcome == "Root Mean Square of Successive Differences", bold = TRUE) %>%

bg(i = ~ Outcome == "Root Mean Square of Successive Differences", bg = "#f2f2f2") %>%

bold(i = ~ Outcome == "Root Mean Square of Successive Differences", bold = TRUE)

\## Combine tables

results_all %>%

mutate(model="primary (40%)") %>%

full_join(results_all60 %>% mutate(model="secondary (60%)")) %>%

full_join(results_all100 %>% mutate(model="secondary (complete case)")) %>%

print(n=50)

\# Combine your results into one tidy table

results_combined <- bind_rows(

results_all	%>% mutate(analysis = "Primary (40%)"),

results_all_sms %>% mutate(analysis = "Primary (40%)"),

results_all60	%>% mutate(analysis = "Secondary (60%)"),

results_all_sms60 %>% mutate(analysis = "Secondary (60%)"),

results_all100 %>% mutate(analysis = "Secondary (Complete Case)")

) %>%

mutate(

outcome = recode(outcome,

rmssd = "Root Mean Square of Successive Differences",

sd_pain = "Standard Deviation of Weekly Pain Intensity",

rmse_trend = "Root Mean Square Error of Residuals",

masd = "Mean Absolute Successive Differences",

pac1 = "Proportion of Weeks with ≥1-Point Change",

pac2 = "Proportion of Weeks with ≥2-Point Change",

n_missing = "Weeks with missing data"),

estimate_CI = sprintf("%.3f (%.3f, %.3f)", estimate, conf.low, conf.high),

summary = paste0(estimate_CI, "\n(p = ", p.value, ")")

) %>%

select(outcome, model, analysis, summary) %>%

pivot_wider(names_from = analysis, values_from = summary) %>%

arrange(factor(outcome, levels = c(

"Root Mean Square of Successive Differences",

"Standard Deviation of Weekly Pain Intensity",

"Mean Absolute Successive Differences",

"Root Mean Square Error of Residuals",

"Proportion of Weeks with ≥1-Point Change",

"Proportion of Weeks with ≥2-Point Change",

"Weeks with missing data"

)),

model)

\# Build flextable

ft_sensitivity <- results_combined %>%

flextable() %>%

set_header_labels(

outcome = "Outcome",

model = "Model",

`Primary (40%)` = "Primary (40%)",

`Secondary (60%)` = "Secondary (60%)",

`Secondary (Complete Case)` = "Secondary (Complete Case)"

) %>%

merge_v(j = 1) %>% # merge outcome names vertically

align(align = "center", part = "all") %>%

autofit() %>%

fontsize(part = "all", size = 11) %>%

font(part = "all", fontname = "Calibri") %>%

border_remove() %>%

border_outer(part = "all", border = officer::fp_border(color = "black", width = 1)) %>%

border_inner_h(part = "body", border = officer::fp_border(color = "gray", width = 0.5)) %>%

border_inner_v(part = "body", border = officer::fp_border(color = "gray", width = 0.5)) %>%

\# Highlight the primary outcome

bg(i = ~ outcome == "Root Mean Square of Successive Differences", bg = "#f2f2f2") %>%

bold(i = ~ outcome == "Root Mean Square of Successive Differences", bold = TRUE)

ft_sensitivity

save_as_docx(ft_sensitivity,path = "A_Papers/Nested_RCT/B_FIGURES_TABLES/regression_sensitivity.docx")
